# Neoadjuvant therapy with peptide receptor radionuclide therapy for pancreatic neuroendocrine tumours

**DOI:** 10.1093/bjs/znae183

**Published:** 2024-08-30

**Authors:** Julie Hallet, Kjetil Søreide

**Affiliations:** Department of Surgery, University of Toronto, Toronto, Ontario, Canada; Division of Surgical Oncology, Susan Leslie Clinic for Neuroendocrine Tumours – Odette Cancer Centre, Sunnybrook Health Sciences Centre, Toronto, Ontario, Canada; Department of Gastrointestinal Surgery, Stavanger University Hospital, Stavanger, Norway; Gastrointestinal Translational Research Group, Laboratory for Molecular Medicine, Stavanger University Hospital, Stavanger, Norway; Department of Clinical Medicine, University of Bergen, Bergen, Norway

**Fig. 1:**
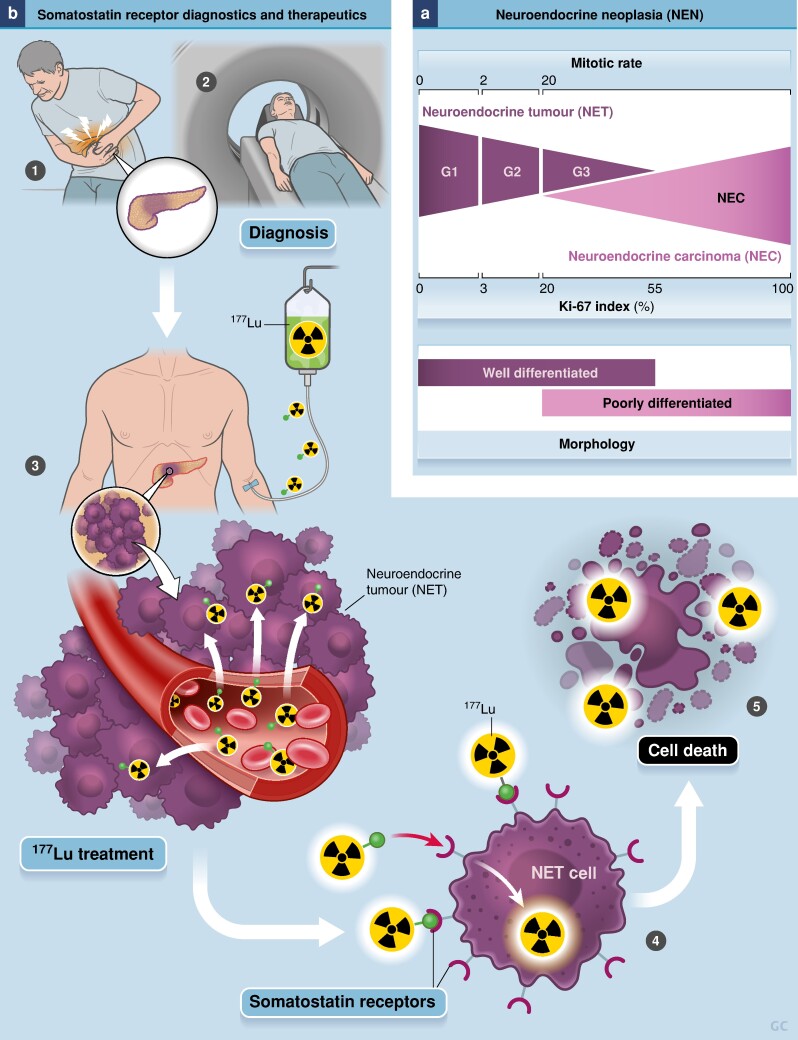
Pancreatic neuroendocrine tumours: grading and treatment **a** Grading of neuroendocrine tumours based on mitotic rate or Ki-67 index. **b** Diagnostic and therapeutic (‘theranostic’) use of somatostatin receptors for neuroendocrine tumours. Somatostatin receptors can be used for both diagnostic purposes (such as PET imaging with ^68^Ga-radiolabelled DOTATATE) and therapeutic purposes (peptide receptor radionuclide therapy (PRRT), also known as receptor–ligand therapy).

Pancreatic neuroendocrine tumours (pNETs) account for close to 10% of all neuroendocrine neoplasms and have worse outcomes compared with other neuroendocrine neoplasms, with reported recurrence-free survival of 37% and overall survival of 30% at 10 years^1^. pNETs are a heterogeneous group of malignancies whose behaviour and outcomes vary significantly depending on tumour grade (*Fig. 1a*), size, and presentation. Most pNETs do not produce hormones (such as insulin, glucagon, or gastrin) and are hence called non-functional pNETs (NF-pNETs). Most of these NF-pNETS are low grade (G1 or G2) (*Fig. 1a*) and resection is recommended when greater than 2 cm in operable patients. While smaller NF-pNETs (less than or equal to 2 cm) can be safely observed with active surveillance, surgical resection is the cornerstone of curative-intent therapy for larger localized and locoregional pNETs^2,3^. Recent decades have seen considerable advancements and changes in the systemic management of patients with advanced or unresectable pNETs. One unique option for systemic therapy for NETs is peptide receptor radionuclide therapy (PRRT). NET cells are characterized by the expression of somatostatin receptors that can be used for ‘theranostic’ approaches, which tie diagnosis and therapy together through molecules that have both imaging and therapeutic abilities. For NETs, this involves somatostatin receptor PET imaging (such as with ^68^Ga-radiolabelled DOTATATE or ^64^Cu-radiolabelled DOTATATE) and PRRT (such as with intravenous ^177^Lu-radiolabelled DOTATATE) (*Fig. 1b*). Despite progress with regard to systemic therapy for NETs, including PRRT, there is no clear evidence to guide perioperative therapy for patients with resectable disease.

Preoperative therapy for NF-pNETs has rarely been investigated and is not currently well defined. While often discussed anecdotally at scientific conferences, preoperative therapy has not been investigated in a systematic way. One would think that the advent of new effective systemic therapies may lead to opportunities for perioperative therapy. In strict terms, neoadjuvant treatment is given before surgery, as an alternative to or in addition to adjuvant treatment after surgery. This may be done to test tumour biology (‘test of time’) before surgery for disease that is known to be aggressive and with a high risk of early recurrence, in which case therapy promoting tumour stability is needed. Neoadjuvant therapy may also be given to facilitate the delivery of multimodal therapy for improved survival, such as in situations with a high risk of postoperative complications that may delay or prevent the receipt of adjuvant treatment after surgery, in which case effective adjuvant therapies options are needed. Finally, preoperative therapy may be used to down-stage tumours to convert locally advanced unresectable disease to resectable disease or to lessen the extent of resection for locally advanced or borderline resectable disease, in which cases therapy with a clinically relevant response rate is needed. Overall, the desirability and feasibility of neoadjuvant or preoperative treatment approaches are dependent on the systemic therapy options available and how they may achieve those goals.

Identifying opportunities for preoperative therapies has so far been based on data from trials for advanced and metastatic pNETs, from which the potential for neoadjuvant and preoperative use has largely been extrapolated. Most therapies have been studied in the second-line setting (and beyond), with only two regimens being examined in the first-line setting, namely lanreotide, showing improved time to progression compared with placebo in the CLARINET trial, and PRRT with ^177^Lu-radiolabelled DOTATATE, showing improved progression-free survival compared with dose escalation of long-acting somatostatin analogues^4,5^. Most existing systemic therapies for pNETs lead to improved time to progression or progression-free survival in trials for advanced and/or metastatic NETs, including lanreotide, targeted therapy with everolimus, sunitinib, lenvantinib, or cabozantinib, cytotoxic chemotherapy with capecitabine/temzolomide, and radio-ligand therapy with ^177^Lu-radiolabelled DOTATATE^4,6–11^. Reported response rates have traditionally been low for most of these therapies, such as everolimus and sunitinib (less than 10%) and long-acting somatostatin analogues (less than 5%). More recently, capecitabine/temzolomide, lenvatinib, cabozantinib, and ^177^Lu-radiolabelled DOTATATE have shown response rates from 30% to 40%^4–11^. However, not much is known about the details of the response: is the response mostly connected to the primary tumour or the metastases? Does the response lead to sufficient down-staging or conversion to change resectability? Finally, no systemic therapy has been shown to impact overall survival or demonstrated efficacy in the adjuvant setting for pNETs at this time.

Experience with the use of neoadjuvant therapy for pNETs has been limited to single-centre small cohort studies^12–14^. Many have anecdotally cited the merits of capecitabine/temzolomide as neoadjuvant therapy in scientific fora, but those retrospective experiences are yet to be published. Regarding the NEOLUPANET study published in *BJS*, Partelli *et al*.^15^ present the first prospective assessment of neoadjuvant therapy for pNETs, if not the first ever prospective assessment of any perioperative therapy for pNETs. This prospective trial is an accomplishment in and of itself, considering the heterogeneity and uncommon nature of pNETs, the difficulties in multi-institutional surgical collaborations in this field dominated by single-centre experiences, and the challenges associated with funding (for resectable disease) and conducting high-quality surgical trials. Beyond outcome data, the surgical community has much to learn from this work.

Retrospective studies of patients treated with PRRT followed by surgery for oligometastatic (less than 3 liver metastases) or potentially resectable high-risk NF-pNETs (such as Ki-67 index greater than 10%, size greater than 4 cm, invasion into nearby organs, or vascular invasion) have reported reductions in tumour size and pathological tumour response^13,14^. The phase II trial of Partelli *et al*.^15^ provides objective prospective data for PRRT followed by surgery for potentially resectable high-risk pNETs. The NEOLUPANET trial enrolled 31 patients, over 3 years at 8 institutions in Italy, to undergo 4 cycles of neoadjuvant ^177^Lu-radiolabelled DOTATATE. The results provide three main findings: they demonstrate a radiological response rate of 59% for localized pNETs; they confirm the ability to generate a response in the primary pNET, with 36% having fibrosis in the specimen; and they establish the safety of resection after PRRT, with rates of postoperative major morbidity and mortality on a par with contemporary outcomes for complex pancreatectomy for cancer. Of note, postoperative pancreatic fistulae were observed in 37% of patients, as can be expected for pancreatectomy for pNETs due to an often softer gland and smaller pancreatic duct than what is found for other malignancies. Another interesting observation was that 7 patients had positive nodal uptake according to preoperative ^68^Ga-radiolabelled DOTATATE PET-CT, while 15 patients had positive nodal disease according to pathology. This demonstrates the challenges associated with identifying the true extent of disease using preoperative imaging and the potential need for perioperative systemic therapy for high-risk resectable pNETs. Future work will be needed to compare effectiveness in terms of long-term oncological outcomes to know whether preoperative PRRT can reduce recurrence and prolong survival.

While this work may not settle the debate on neoadjuvant therapy for pNETs, it represents an important stake in the sand and sets the stage for much work to come in the perioperative management of pNETs. Additional work is warranted to define which systemic therapy is best in the neoadjuvant setting, how response rates translate into meaningful down-staging and resectability when needed, and whether there is an impact on long-term survival outcomes. Preoperative PRRT with ^90^Y-radiolabelled DOTATOC is currently the subject of another phase II single-arm trial (NeoNet trial, ClinicalTrials.gov NCT05568017) for patients with unresectable or borderline resectable pNETs and limited liver disease. For the time being, there are some important takeaways from the NEOLUPANET data. First, PRRT is a safe perioperative therapy that can be examined formally in comparative effectiveness trials to improve cancer control for resectable pNETs at higher risk of recurrence. Second, it is a promising strategy that can be used for down-staging locally advanced disease to resectable disease or to less-extensive resections. Third, surgical clinical trials can be conducted for pNETs with multi-institutional collaboration. Awaiting other data, neoadjuvant therapy with PRRT can be considered for selected pNETs, knowing it leads to considerable response rates and does not adversely affect postoperative outcomes.
